# What Does Our Personality Say About Our Dietary Choices? Insights on the Associations Between Dietary Habits, Primary Emotional Systems and the Dark Triad of Personality

**DOI:** 10.3389/fpsyg.2019.02591

**Published:** 2019-11-22

**Authors:** Rayna Sariyska, Sebastian Markett, Bernd Lachmann, Christian Montag

**Affiliations:** ^1^Department of Molecular Psychology, Institute of Psychology and Education, Ulm University, Ulm, Germany; ^2^Department of Psychology, Humboldt-Universität zu Berlin, Berlin, Germany

**Keywords:** personality, ANPS, Machiavellianism, narcissism, psychopathy, diet

## Abstract

The awareness of the consequences of consuming animal products for the environment and one’s own health has been growing in recent years. The aim of the present research project was to examine the relationship between individual differences in biologically rooted primary emotional systems arising from phylogenetically old brain areas and dietary habits including being a vegan/vegetarian or omnivore (Study 1). Additionally, the link between the Dark Triad personality traits and dietary habits was investigated (also Study 1). In Study 2 it was aimed to replicate the associations between the Dark Triad traits and dietary habits in a new sample. In total 1140 (Study 1) and 444 (Study 2) participants took part in the research project. The Affective Neuroscience Personality Scales (ANPS) were applied to assess individual differences in six primary emotional systems. The Short Dark Triad Scale (SD3) was administered to assess individual differences in Machiavellianism, psychopathy and narcissism. The eating style of participants was measured with the Eating Behavior Questionnaire (EBQ). Results of Study 1 demonstrated higher CARE, SADNESS and spirituality scores, and lower PLAY scores in vegans/vegetarians than in omnivores. However, after the sex of the participants was included in the model, the effect on CARE got weaker. Additionally, omnivores scored higher on Machiavellianism, however, this association disappeared when sex was added to the model. In Study 2, higher scores in Machiavellianism, narcissism and psychopathy were reported for the group of omnivores compared to vegans/vegetarians, however, those effects got weaker or disappeared after the sex of participants was added to the model. The present research project adds to the literature by investigating the ANPS model and the Dark Triad of personality in the context of eating style for the first time. The findings of these two studies might help to better understand how people following different types of diet, might differ in their personalities.

## Introduction

The number of consumers following a plant-based diet has dramatically increased in recent years (The Guardian, 2018). Vegan and vegetarian diets attract the attention for different reasons such as health, weight loss, ethical, environmental or religious purposes to name a few (Janssen et al., 2016). The manifold reasons to endorse such a diet might also vary depending on the cultural background or sex of individuals, as well as social norms (Ruby, 2012).

One commonly used definition describes vegetarianism on a scale ranging from *least strict* to *most strict* resulting in six types (Beardsworth and Keil, 1992). The first type includes people characterizing themselves as vegetarians, although they occasionally consume meat. While the second type vegetarians avoid the consumption of meat, the third type also excludes fish and the forth type also eggs from their diet. Type five vegetarians avoid additionally the consumption of dairy products, while type six vegetarians (also called vegans) consumes solely plant-based foods. In a representative study from Germany, 2.74% of the investigated participants reported to be vegetarians who avoid the consumption of meat and fish (Pfeiler and Egloff, 2018a).

In Western societies, sex differences regarding attitudes and beliefs with respect to dietary habits have been demonstrated. Women express more favorable attitude toward animals, while men believe that meat consumption makes them “manlier” (Rothgerber, 2013). These attitudes are mirrored in statistics, showing that vegetarian women outnumber vegetarian men (see Ruby, 2012). Additionally, higher meat consumption has been linked to beliefs in human’s supremacy and the perception of vegetarianism as a threat for traditional dietary choices in Leite et al. (2018). Furthermore, associations between veganism and/or vegetarianism and higher empathy (e.g., Preylo and Arikawa, 2008; Filippi et al., 2010), as well as between meat consumption and political ideology (e.g., conservatism) have been shown (Hodson and Earle, 2018).

Next to the variables listed above, individual differences in personality might also play a significant role in explaining one’s own food choices. In this context, the Five-factor model of personality has been often investigated. Here, high openness for experiences seems to be a rather robust predictor of vegetarian food choices (e.g., Forestell and Nezlek, 2018; Pfeiler and Egloff, 2018a, b). However, also associations between openness and meat consumption, which vary depending on the type of meat to be consumed, have been reported (Pfeiler and Egloff, 2018c). The associations with respect to conscientiousness and meat consumption vary, where positive and negative relationships have been reported in different studies (e.g., Pfeiler and Egloff, 2018a, b). Extraversion has been linked to higher meat consumption (Pfeiler and Egloff, 2018c), neuroticism to a higher probability of having a vegetarian diet, whereas studies exploring associations between agreeableness and dietary style demonstrated mixed results (e.g., Forestell and Nezlek, 2018; Pfeiler and Egloff, 2018b). It also needs to be mentioned that there are studies which failed to find significant differences between vegans/vegetarians and omnivores with respect to the Big-Five personality traits (e.g., Kessler et al., 2018).

Aside from the Five-factor model of personality, to our knowledge, only a few other personality traits have been tested in the context of dietary choices. However, since the consumption of food is necessary in order to survive and is, thus, of high evolutional significance, it is of high interest to investigate biologically based models of personality going beyond the Five-factor model to get further insights into eating style.

Panksepp (2004) administered deep brain stimulation and pharmacological challenges in the mammalian brain and discovered seven primary emotional systems which shape humans’ behavior and personality in a bottom up fashion (Davis et al., 2003; Montag and Panksepp, 2017; Davis and Montag, 2018; Montag and Davis, 2018). Those emotional systems have been linked to specific subcortical areas in the mammalian brain and to distinct neurotransmitter systems (Panksepp, 2004, 2005). These ancient emotional systems can be grouped into positive affect (SEEKING, CARE, PLAY, and LUST^1^) and negative affect (ANGER, FEAR, SADNESS). Compared to the Five-factor model that has been developed based on a lexical approach, Panksepp’s Affective Neuroscience theory places human’s personality on a strong biological fundament (Montag and Davis, 2018). The investigation of primary emotional systems in the context of eating behavior is also of importance, because individual differences in primary emotional systems could be the fundament of the Big Five of Personality (perhaps with the exception of conscientiousness; see Montag and Panksepp, 2017). Thus, next to the evolutionary importance of both, nutrition and the primary emotional systems, another link that connects them are studies examining the associations between diet and personality. Since it has been demonstrated that both, the primary emotional systems (Davis and Panksepp, 2011) and diet, are linked to the Five-factor model, such studies might help to further narrow down the focus on particular primary emotional systems to describe food choices.

Probably the most obvious candidate from the primary emotional systems to be linked to the vegan/vegetarian diet is CARE. People with a highly active CARE system can be described as strongly feeling empathy and liking to care for others, including pets and children (Davis et al., 2003). Given this definition and the overlap between CARE and empathy, it is proposed that CARE might explain some of the variance in vegetarian food choices. In line with this idea, the vegan/vegetarian diet has been linked to higher empathy compared to the omnivorous diet in numerous studies (e.g., Preylo and Arikawa, 2008; Filippi et al., 2010; Rothgerber, 2015). Of note, vegans were shown to report even higher levels in empathy than vegetarians (Kessler et al., 2016). High CARE (together with low ANGER) might represent the evolutionary basis for agreeableness (Montag and Davis, 2018). Despite mixed results reported on the link between the vegetarian diet and agreeableness, the investigation of CARE is of high relevance in the context of eating behavior.

As openness to experiences has often been linked to both a plant-based diet and SEEKING (being curious, being drawn toward new experiences; Davis et al., 2003; Davis and Panksepp, 2011), it is likely that individual differences in SEEKING might play an important role in the context of food choices, too. The associations between dietary choices and FEAR, SADNESS and ANGER as well as spirituality will be examined exploratory, since the existing literature does not allow for more specific hypotheses at this point. In general, the negative emotional systems FEAR, SADNESS, and ANGER are known to be tightly linked to high neuroticism, and the latter has been associated with vegetarianism (see Davis and Panksepp, 2011; Forestell and Nezlek, 2018). However, since neuroticism is a rather widely defined concept, compared to the more narrowly defined emotional systems of FEAR, SADNESS and ANGER, it is not clear what associations could be expected with food choices. For example, ANGER, which is related to aggression, might be linked to an omnivorous diet rather than a vegan/vegetarian diet (see Davis et al., 2003; Jain et al., 2018). Last but not least, spirituality, described as searching for the meaning in life and feeling connected to the world and creation as a whole (Davis et al., 2003; Davis and Panksepp, 2011), per definition might be rather linked to the vegan/vegetarian diet.

On another note, associations between beliefs in human’s supremacy (Leite et al., 2018) or hierarchical domination (in specific right-wing authoritarianism and social dominance orientations) (Allen et al., 2000) and the omnivorous diet draw the attention toward another personality model, which has not been investigated in the context of dietary choices so far: The Dark Triad of personality. Here, the traits Machiavellianism, non-pathological narcissism and non-pathological psychopathy have been put forward (Paulhus and Williams, 2002). Machiavellianism describes a manipulative personality which lacks morality, and has a focus on self-interest. Narcissism includes grandiosity, entitlement, dominance and superiority. Lastly, psychopathy in terms of the Dark Triad includes high impulsivity, thrill-seeking going along with low empathy, and with antisocial behavior (Paulhus and Williams, 2002; Jones and Paulhus, 2014). The Dark Triad traits are intercorrelated and are assumed to constitute the basis of a higher-order trait. However, each of the traits also exhibits its own unique features (Rauthmann, 2012; Muris et al., 2017). Common correlates of the Dark Triad traits are low agreeableness, low honesty-humility (HEXACO), low empathy and high alexithymia (Furnham et al., 2013; Schimmenti et al., 2019). With respect to the link with empathy, differences between the Dark Triad traits were demonstrated, where psychopathy was negatively linked to perspective taking, fantasy and empathic concern, whereas narcissism was positively associated with fantasy and personal distress (Jonason and Kroll, 2015). Those positive associations between narcissism and empathy imply that understanding the thoughts and feelings of others might be crucial for narcissists in order to receive validation for their ego-needs (Jonason and Kroll, 2015). A recent meta-analysis demonstrated that the Dark Triad traits, and especially Machiavellianism and psychopathy, are linked to aggression, interpersonal problems and antisocial strategies (Muris et al., 2017). In this context, psychopathy and Machiavellianism are described as more negative traits, whereas the behavior of people high in narcissism has been rated by others as rather neutral (Rauthmann, 2012). From an evolutionary perspective, mating issues have been linked to the Dark Triad traits. Here, studies have demonstrated that the Dark Triad traits are linked to a fast-life history strategy, thus, accentuating mating compared to parenting. This strategy is associated with short-term mating, antisocial behavior and low self-control (Jonason et al., 2009; Furnham et al., 2013).

The link between high empathy and the vegetarian diet, reported above indicates a possible positive association between the omnivorous diet and the Dark Triad traits, the latter being associated with lower empathy. Additionally, an association between hierarchical justification for meat eating (e.g., “Humans are at the top of the food chain and meant to eat animals.,” Rothgerber, 2013, p. 13) and higher meat consumption and lower consumption of vegetarian dishes points toward a possible link between narcissism and omnivorous dietary choices. Recent research also demonstrated an association between aggression and the omnivorous diet (Jain et al., 2018). With respect to the link between the Dark Triad traits and antisocial behavior reported above, as well as the link between the Dark Triad traits and schadenfreude (experiencing pleasure, when others are suffering or are misfortunate), and dispositional aggression (James et al., 2014; Jones and Neria, 2015), those associations give an additional hint toward the potential relationship between the Dark Triad traits and an omnivorous diet. Last but not least, the Dark Triad traits were associated with more negative attitudes toward animals, and psychopathy was additionally linked to behaviors demonstrating animal cruelty (Kavanagh et al., 2013). This last finding demonstrates that callous behavior and attitudes linked to the Dark Triad traits are not limited to human-to-human interactions, but are also relevant with regard to human-to-animal interactions (Kavanagh et al., 2013). Moreover, the Dark Triad traits are suggested to foster survival from an evolutionary point of view (Furnham et al., 2013). The negative attitudes and behavior toward animals, expressed by people scoring high on the Dark Triad traits, might result in meat consumption, which on the other hand will foster survival. This is another argument for the positive association between the Dark Triad traits and the omnivorous diet.

It is important to note at this point that empirical evidence on sex differences regarding the Dark Triad traits exists. Here, males usually score higher on all Dark Triad traits than females (Muris et al., 2017). The same is true for dietary choices, where more females than males chose a vegan/vegetarian diet (Ruby, 2012). Thus, particular attention will be paid on possible interactions between sex, diet and the Dark Triad traits.

The aim of the present study is to investigate individual differences in primary emotional systems, the three Dark Triad traits and their potential role for dietary choices. The focus of the two studies, described in detail further below, lies on differentiating between vegans/vegetarians (combined to one group) and omnivores with respect to the Affective Neuroscience Personality Framework and the Dark Triad of personality. To the authors’ knowledge these associations have not been investigated before.

## Materials and Methods

### Participants

In Study 1, in total *N* = 1255 volunteers, part of the Ulm Gene Brain Behavior Project, participated. Of note some participants were also included with a totally different focus, namely the Dark Triad traits and Internet Use Disorders in another sample (Sindermann et al., 2018b). Among others, articles have been published with a smaller subsample using the ANPS on revengefulness (Sindermann et al., 2018a) and Internet Use Disorder (Montag et al., 2016). After missing data in all questionnaires, inconsistent responses on the Eating Behavior Questionnaire (EBQ), the pescatarian group and minors (participants under the age of 18) were excluded, the final data set consisted of *N* = 1140 participants (783 females). Participants’ average age was 23.54 (*SD* = 7.06, range 18–82). In total, 0.2% of participants reported having no degree, 0.4% secondary modern school qualification, 3.6% O-level, 2.7% vocational baccalaureate diploma, 72.5% A-levels, 2.8% polytechnic degree, and 17.7% university degree (numbers do not sum up to exactly 100% due to inaccuracies when rounding). Here, 131 (11.5%) participants reported being vegan or vegetarian and 1009 (88.5%) being omnivore (please see the following section for detailed information about data cleaning and assignment to groups). The percentage of vegans and vegetarians in the group of females (14.0%) was higher than in males (5.9%). Additionally, 94.1% of male participants reported being omnivores, compared to 86.0% of female participants. In total, the most common reasons for avoiding meat consumption reported by vegans and vegetarians were ethical reasons (71.8%), disgust (13.0%), and health (4.6%). The remaining participants reported weight reduction, religious reasons or other reasons for not consuming meat.

In total, *N* = 549 volunteers participated in Study 2. Part of this sample has been already used in a previous publication with another thematic focus, namely on the Dark Triad of personality and Internet Use Disorders, already reported above (see Sindermann et al., 2018b). After participants who already took part in Study 1, participants with missing values and those who gave inconsistent responses on the EBQ, pescatarians and minors were excluded, the final dataset resulted in *N* = 444 participants (312 females). The average age of participants was 30.12 (*SD* = 14.61, range 18–83) years. In total 0.2% reported having no degree, 3.4% secondary modern school qualification, 13.1% O-level, 5.6% vocational baccalaureate diploma, 55.2% A-levels, 4.1% polytechnic degree, and 18.5% university degree (numbers do not sum up to exactly 100% due to inaccuracies when rounding). Here, 55 (12.4%) participants reported being vegan or vegetarian and 389 (87.6%) being omnivore. The percentage of vegans and vegetarians in the group of females (16.0%) was higher than in males (3.8%). Additionally, 96.2% of male participants reported being omnivores, compared to 84.0% of female participants. Among the reasons reported by vegan and vegetarian participants for avoiding the consumption of meat, the most common were ethical reasons (78.2%), followed by disgust (10.9%) and health (7.3%). The remaining participants chose the answer “other reasons”.

### Materials

The (EBQ, developed based on Bilewicz et al., 2011, and translated into German) includes three questions, measuring the dietary habits of participants. The first question assesses the diet type. Here, participants can choose between vegan (following a strict plant-based diet, thus, refraining from consuming meat, fish, dairy products and eggs), ovo-lacto-vegetarian (not consuming fish and meat), pescatarian (not consuming meat) or omnivore (not limiting their diet with respect to animal products). The second question was answered only by vegans/vegetarians and pescatarians, and assessed the reasons for avoiding meat consumption (health, disgust, ethical reasons, weight loss, religious reasons, other reasons). Finally, the third question included a list of 10 different food categories (fruits, vegetables, honey, pasta/noodles, potatoes, eggs, fish/sea food, pork, poultry, and red meat) where participants were instructed to rate the frequency with which they consume those foods during a regular week (from 1 = “never” to 7 = “six times per week or more”). Before analyzing the data, it was examined if the reported type of diet according to the first question (e.g., following a vegan diet) corresponded to the responses regarding the frequency of consumption of different types of foods on the third question (e.g., never consuming meat, fish, eggs). Where inconsistencies were found, participants were excluded from the analyses.^2^ Vegan and vegetarian participants were combined to one group to increase the statistical power, because both groups were rather small. For the present study, the pescatarian group was not analyzed as there is a discourse in the literature if pescatarians are better described as vegetarians or omnivores. As mentioned in the section “Participants”, pescatarians were excluded before running the statistical analyses.

*Affective Neuroscience Personality Scales* [ANPS; Davis et al., 2003; German version by Reuter et al. (2017)] consist of 110 questions, rated on a scale from 1 = “strongly disagree” to 4 = “strongly agree”. Six primary emotions can be assessed using this questionnaire: SEEKING, PLAY, CARE, FEAR, ANGER, SADNESS, together with the dimension of spirituality. The possible range for every scale except for spirituality is 14–56. The possible range for the scale spirituality is 12–48. The reliabilities for Study 1 were as follows: SEEKING α = 0.71, FEAR α = 0.87, CARE α = 0.80, ANGER α = 0.84, PLAY α = 0.79, SADNESS α = 0.75, spirituality α = 0.85. The ANPS questionnaire was only filled in by the participants of Study 1.

*The Short Dark Triad* [SD3; Jones and Paulhus (2014)] was developed to assess the traits Machiavellianism, non-pathological narcissism and non-pathological psychopathy. A German translation of this questionnaire was used in the present study. The German version was already applied in a study by Sindermann et al. (2018b), where also the factor structure was assessed. It includes 27 items (nine items per scale), which are rated on a scale from 1 = “disagree strongly” to 5 = “agree strongly.” The possible range for every scale varies between 9 and 45. Even though the SD3 is a short measure of the Dark Triad traits, its validity and reliability has been demonstrated in many studies already (Jones and Paulhus, 2014). The reliabilities of the three scales for the current samples were as follows: Machiavellianism α = 0.78, narcissism α = 0.64, psychopathy α = 0.73 in Study 1, and Machiavellianism α = 0.71, narcissism α = 0.62, psychopathy α = 0.71 in Study 2. In Study 1, SD3-data from 508 participants was available.

### Procedure

Participants were mostly recruited in classes of Psychology at Ulm University, Ulm, Germany. Participants were then contacted via e-mail which included the link to the online-questionnaire. In both studies, participants had the opportunity to save the questionnaire and fill it in later, by using the link sent them per e-mail. In both studies participants were informed about the aim of the study and gave digital informed consent prior to their participation. Both studies were approved by the local ethic committee of Ulm University, Ulm, Germany.

### Statistical Analyses

First, the distribution of variables was tested by assessing the skewness and kurtosis of the variables, where values < 2 were considered as normally distributed (Miles and Shevlin, 2001). Second, associations with age and sex were assessed using Pearson or Spearman correlations, and *t*-test or Mann–Whitney *U* test depending on the distribution of the variables. For non-normally distributed variables, where necessary a Blom rank-based transformation was used for normalization purposes. A MANOVA was conducted to assess the associations between dietary habits and the ANPS, and SD3 scales. Where significant associations between age, sex and the remaining variables were demonstrated, age was added as a control variable into the analyses, while sex was entered as a second independent variable next to the variable diet. This way, it was possible to examine potential interactions between sex and diet on the ANPS and Dark Triad traits. Finally, using Pearson correlation analyses with the Bootstrap BCa 95% CI, the relationship between the frequency of consuming different foods and the ANPS, and SD3 scales was tested (the results of the correlation analyses are presented in the Supplementary Material). These results are also presented separately for male and female participants.

## Results

### Study 1

In the following, first the descriptive statistics of the sample in Study 1 are presented, split in vegans/vegetarians vs. omnivores (see Tables 1, 2). The overall and by sex descriptive statistics are presented in Supplementary Tables S1, S2. After that, the associations between age, sex and the remaining variables was assessed (see section “Associations With Age and Sex”) and the effect of diet on the ANPS and Dark Triad traits was examined (see section “Differences Between Vegans/Vegetarians and Omnivores With Respect to Personality”).

**TABLE 1:** Descriptive statistics for age and the ANPS variables, split in vegan/vegetarian vs. omnivore (Study 1).

		**Age**	**SEEKING**	**FEAR**	**CARE**	**ANGER**	**PLAY**	**SADNESS**	**Spirituality**
Vegan/vegetarian	*N*	131	131	131	131	131	131	131	131
	Mean	22.54	40.23	37.68	43.58	35.44	41.17	36.23	27.46
	Median	21	40	37	44	36	41	36	27
	*SD*	6.14	5.09	7.25	5.60	6.53	5.86	5.95	7.18
	Skewness	3.95	−0.05	0.13	−0.65	0.14	−0.54	0.22	0.16
	Kurtosis	17.99	−0.39	−0.41	0.76	0.30	0.93	0.61	−0.71
	Min.	18	25	19	24	21	19	20	16
	Max.	57	51	54	55	56	54	54	45
Omnivore	*N*	1009	1009	1009	1009	1009	1009	1009	1009
	Mean	23.67	39.64	36.63	41.05	35.92	42.23	34.59	25.24
	Median	22	40	36	41	36	42	34	25
	*SD*	7.17	4.36	6.58	5.86	6.14	5.52	5.23	6.32
	Skewness	3.55	−0.07	−0.00	−0.34	0.07	−0.30	0.18	0.26
	Kurtosis	14.72	0.73	−0.30	0.18	0.13	0.23	−0.04	−0.13
	Min.	18	20	17	16	17	21	22	12
	Max.	82	53	54	56	55	56	52	47

**SD* = standard deviation, N = number of participants.*

**TABLE 2:** Descriptive statistics for age and the SD3 variables, split in vegan/vegetarian vs. omnivore (Study 1).

	**Vegan/vegetarian**				**Omnivore**			
	**Age**	**Machiavellianism**	**Narcissism**	**Psychopathy**	**Age**	**Machiavellianism**	**Narcissism**	**Psychopathy**
*N*	70	70	70	70	438	438	438	438
Mean	23.37	24.94	22.49	17.74	23.48	26.65	23.57	18.75
Median	21	25	23	17	21	27	23	19
*SD*	8.00	5.69	4.65	5.06	8.62	5.70	4.24	5.12
Skewness	3.10	0.18	−0.36	0.61	3.39	0.05	0.11	0.53
Kurtosis	9.76	−0.29	−0.50	0.22	12.27	0.06	0.02	0.41
Min.	18	13	12	9	18	12	11	9
Max.	57	39	31	34	82	44	38	38

**SD* = standard deviation, N = number of participants.*

#### Associations With Age and Sex

All ANPS and SD3 Scales were normally distributed. Age was non-normally distributed (Miles and Shevlin, 2001). Spearman correlations revealed that age was significantly associated with FEAR (ρ = −0.11, *p* < 0.01), CARE (ρ = −0.09, *p* < 0.01), PLAY (ρ = −0.13, *p* < 0.01), SADNESS (ρ = −0.10, *p* < 0.01), Machiavellianism (ρ = −0.19, *p* < 0.01), and narcissism (ρ = −0.16, *p* < 0.01). *T*-tests revealed significant sex differences regarding the ANPS and SD3 scales. Here, female participants scored higher on FEAR, CARE, ANGER, SADNESS, and spirituality (*p* < 0.01; see Supplementary Table S1). Moreover, male participants scored higher than females on Machiavellianism, narcissism, and psychopathy (*p* < 0.01; see Supplementary Table S2). Vegans/vegetarians and omnivores significantly differed in age in the complete sample (*Z* = −2.723, *p* < 0.01; see Table 1 for the means of both groups), and dietary habits were significantly linked to sex [*X*^2^(1) = 16.077, *p* < 0.01]. Thus, sex and age were considered in the following analyses.

#### Differences Between Vegans/Vegetarians and Omnivores With Respect to Personality

A MANOVA revealed significant differences between vegans/vegetarians and omnivores with respect to the ANPS scales [Pillai’s Trace: *F*(7, 1132) = 6.950, *p* < 0.001, ${\text{η}}_{p}^{2}$ = 0.04]. Here, the scales CARE [*F*(1, 1138) = 21.761, *p* < 0.001, ${\text{η}}_{p}^{2}$ = 0.02], SADNESS [*F*(1, 1138) = 11.037, *p* = 0.001, ${\text{η}}_{p}^{2}$ = 0.01], spirituality [*F*(1, 1138) = 13.855, *p* < 0.001, ${\text{η}}_{p}^{2}$ = 0.01], and PLAY [*F*(1, 1138) = 4.202, *p* = 0.041, ${\text{η}}_{p}^{2}$ = 0.004] reached significance in the univariate tests. Higher scores in CARE, SADNESS, spirituality and lower scores in PLAY were observed in vegans/vegetarians compared to omnivores (see Table 1 for the means of the variables). After a Bonferroni correction for multiple testing was applied (*p* = 0.05/7 = 0.007), all associations except the one with PLAY remained significant. After entering “age” (Blom transformed) as a covariate in the analysis, the results did not change, thus, significant results from the previous analysis remained significant. In the next step, the sex of participants was entered as a second independent variable in order to test for interactions between the dietary style and sex (here age was not included as a covariate because it did not change the associations between dietary style and the primary emotional systems as demonstrated in the previous analysis). The multivariate test for the interaction effects did not reach significance [Pillai’s Trace: *F*(7, 1130) = 1.412, *p* = 0.197]. Regarding the univariate tests, only the interaction between sex and dietary style on SADNESS reached significance [*F*(1, 1136) = 6.382, *p* = 0.012, ${\text{η}}_{p}^{2}$ = 0.01], however, this effect would not remain significant after a Bonferroni correction is applied (*p* = 0.05/7 = 0.007). Moreover, please note that the subsample of vegan/vegetarian male participants included only 21 people, thus, limiting the power for this type of analyses. The univariate tests for the main effects of sex were significant for FEAR (*p* = 0.008, ${\text{η}}_{p}^{2}$ = 0.01), CARE (*p* < 0.001, ${\text{η}}_{p}^{2}$ = 0.06) and SADNESS (*p* = 0.003, ${\text{η}}_{p}^{2}$ = 0.01) before a Bonferroni correction. Regarding diet, the univariate tests after including sex as a second independent variable remained significant for CARE (*p* = 0.048, ${\text{η}}_{p}^{2}$ = 0.003), PLAY (*p* = 0.027, ${\text{η}}_{p}^{2}$ = 0.004), SADNESS (*p* = 0.001, ${\text{η}}_{p}^{2}$ = 0.01), and spirituality (*p* = 0.005, ${\text{η}}_{p}^{2}$ = 0.01) before a Bonferroni correction (see group means in Table 1).

Another MANOVA was conducted to compare the SD3 scores among the investigated groups. Here, although descriptively the scores on all three scales were higher in omnivores, results did not reach significance [Pillai’s Trace: *F*(3, 504) = 2.324, *p* = 0.074). On an univariate level omnivores and vegans/vegetarians differed in Machiavellianism [*F*(1, 506) = 5.439, *p* = 0.020, ${\text{η}}_{p}^{2}$ = 0.01]. However, this result would not hold a correction for multiple testing (*p* = 0.05/3 = 0.017). The results slightly changed after “age” (Blom transformed) was added as a covariate in the analyses. On a univariate level, Machiavellianism (*p* = 0.017, ${\text{η}}_{p}^{2}$ = 0.01) and narcissism (*p* = 0.046, ${\text{η}}_{p}^{2}$ = 0.01) reached significance before a Bonferroni correction (see Table 2 for means in both groups). After sex was added as a second independent variable into the analysis (without age as a covariate), no significant multivariate effect for the interaction between sex and the dietary style could be observed [Pillai’s Trace: *F*(3, 502) = 1.166, *p* = 0.322]. The same was true for the univariate effects regarding this interaction. The univariate main effects of dietary style for the Dark Triad traits did not reach significance, whereas sex had a significant effect on the Dark Triad traits Machiavellianism (*p* = 0.001, ${\text{η}}_{p}^{2}$ = 0.02), narcissism (*p* = 0.038, ${\text{η}}_{p}^{2}$ = 0.01) and psychopathy (*p* < 0.001, ${\text{η}}_{p}^{2}$ = 0.05) before a Bonferroni correction. Please note that the male vegan/vegetarian group included only 10 participants.

Next, the Bootstrap BCa 95% CI Pearson correlations between the frequency of consumption of different foods and the ANPS and Dark Triad traits were calculated (see Supplementary Tables S3, S5). Because the variables “honey,” “red meat” and “fish/seafood” deviated from the normal distribution (Miles and Shevlin, 2001), we also report the Spearman correlations for these variables in the Supplementary Material (Supplementary Table S7).

The results of the correlation analysis revealed some positive correlations between SEEKING, CARE, spirituality and the frequency of consumption of fruits and vegetables. CARE, SADNESS, FEAR, and spirituality were negatively associated with the consumption of red meat and pork (see Supplementary Table S3). Machiavellianism, narcissism and psychopathy were positively linked to the consumption of poultry and red meat. Additionally, narcissism was positively linked to the consumption of fish/seafood and eggs, while Machiavellianism and psychopathy were positively linked to the consumption of pork (see Supplementary Table S5). As the sex of participants was linked to the ANPS and Dark Triad traits, we report the correlation analyses separately for male and female participants in the Supplementary Material (Supplementary Tables S4, S6). Interestingly, only the correlation between psychopathy and poultry consumption remained significant in the male subsample, whereas most of the reported associations between the Dark Triad traits and the frequency of consumption of different foods for the complete sample remained significant in the female subsample.

To our knowledge the ANPS has never been investigated in the context of the Dark Triad personality before. For future research and for the reasons of completeness, we also provide the correlations between these inventories in Table 3. The results of this analysis demonstrated a significant negative link between CARE and all three Dark Triad traits. However, please note that the correlation between CARE and narcissism was rather weak. Moreover, ANGER was positively linked to Machiavellianism and psychopathy. Thus, CARE and ANGER showed the most consistent associations with the Dark Triad traits.

**TABLE 3:** Pearson’s correlations for the associations between the ANPS scales and the Dark Triad traits (Study 1).

	**SEEKING**	**FEAR**	**CARE**	**ANGER**	**PLAY**	**SADNESS**	**Spirituality**
Machiavellianism	0.03	0.12^∗∗^	–0.26^∗∗^	0.28^∗∗^	–0.05	0.09	–0.12^∗∗^
Narcissism	0.26^∗∗^	–0.29^∗∗^	−0.09^∗^	0.07	0.20^∗∗^	–0.24^∗∗^	0.21^∗∗^
Psychopathy	0.05	–0.03	–0.31^∗∗^	0.39^∗∗^	–0.04	0.05	–0.02

**N* = 508, ^∗^*p* < 0.05, ^∗∗^*p* < 0.01, two–tailed tests.*

### Study 2

In the following, the results from Study 2 will be reported, starting with the descriptive statistics (Table 4 and Supplementary Table S8) and after that assessing the relationship between diet, the SD3 scales, age and sex (see section “Associations With Age and Sex” and “Differences With Respect to Personality”).

**TABLE 4:** Descriptive statistics for age and the SD3 scales, split in vegan/vegetarian vs. omnivore (Study 2).

	**Vegan/vegetarian**				**Omnivore**			
	**Age**	**Machiavellianism**	**Narcissism**	**Psychopathy**	**Age**	**Machiavellianism**	**Narcissism**	**Psychopathy**
*N*	55	55	55	55	389	389	389	389
Mean	23.93	21.55	20.38	15.44	31.00	24.40	23.25	17.35
Median	21	21	20	15	23	24	23	16
*SD*	9.09	5.09	4.18	4.34	15.04	5.07	4.17	5.00
Skewness	2.61	0.20	0.54	1.17	1.26	0.05	0.18	0.65
Kurtosis	5.90	−0.03	0.08	1.35	0.44	−0.05	0.03	−0.18
Min.	18	10	13	9	18	10	12	9
Max.	57	33	33	28	83	41	37	32

*The raw data is available as a Supplementary Material (“Raw data Study 2”). *SD* = standard deviation, N = number of participants.*

#### Associations With Age and Sex

Machiavellianism, narcissism and psychopathy were normally distributed. Age was non-normally distributed only in the group of vegans/vegetarians (Miles and Shevlin, 2001). The group of vegans/vegetarians was significantly younger than the group of omnivores (*Z* = −3.794, *p* < 0.001, means for each group are depicted in Table 4). Age was not correlated with the SD3 scales (*p* > 0.05). The sex of participants was linked to their dietary style [*X*^2^(1) = 12.800, *p* < 0.001] and to the SD3 scales, where male participants reported higher values in Machiavellianism, narcissism and psychopathy than female participants (*p* < 0.01; see Supplementary Table S8). As only sex was associated with both the EBQ and the SD3 scales, only sex was entered as a second independent variable next to diet in the following analyses.

#### Differences With Respect to Personality

The results of a MANOVA revealed significant mean differences between vegans/vegetarians and omnivores with respect to the SD3 scales [Pillai’s Trace: *F*(3, 440) = 9.931, *p* < 0.001, ${\text{η}}_{p}^{2}$ = 0.06]. The univariate tests demonstrated that omnivores scored higher on all three scales: Machiavellianism [*F*(1, 442) = 15.241, *p* < 0.001, ${\text{η}}_{p}^{2}$ = 0.03], narcissism [*F*(1, 442) = 22.736, *p* < 0.001, ${\text{η}}_{p}^{2}$ = 0.05] and psychopathy [*F*(1, 442) = 7.245, *p* = 0.007, ${\text{η}}_{p}^{2}$ = 0.02]. Thus, these results would remain significant after a Bonferroni correction is applied (*p* = 0.05/3 = 0.017). In a next step, we added sex as a second independent variable in the MANOVA. The multivariate effect for the interaction between sex and diet did not reach significance [Pillai’s Trace: *F*(3, 438) = 0.824, *p* = 0.481]. The same was true for the univariate effects for these interactions. However, please note that the number of male vegans/vegetarians was 5, thus, reducing the power of the analysis. Regarding the univariate main effects, sex was linked to narcissism (*p* = 0.037, ${\text{η}}_{p}^{2}$ = 0.01) and psychopathy (*p* = 0.030, ${\text{η}}_{p}^{2}$ = 0.01) before a Bonferroni correction, whereas diet showed an effect on Machiavellianism (*p* = 0.010, ${\text{η}}_{p}^{2}$ = 0.02) and narcissism (*p* = 0.029, ${\text{η}}_{p}^{2}$ = 0.01) also before a Bonferroni correction.

Furthermore, Pearson correlations with the Bootstrap BCa 95% CI were used to test the link between the frequency of consumption of different foods and the SD3 scales (see Supplementary Table S9). Here, significant positive correlations were observed between all SD3 scales and the consumption of pork, eggs, red meat, fish/sea food and poultry. Negative correlations were observed with fruits and vegetables (here only the correlation between narcissism and vegetable consumption did not reach significance). Since the variable “frequency of consumption of red meat” deviated from the normal distribution (Miles and Shevlin, 2001), here we report the Spearman correlations for Machiavellianism ρ = 0.20, *p* < 0.001, narcissism ρ = 0.21, *p* < 0.001 and psychopathy ρ = 0.20, *p* < 0.001.

Next, we split the analysis by considering the sex of participants (see Supplementary Table S10). Here, also the Pearson correlations with the Bootstrap BCa 95% CI are reported. Interestingly, in the group of males only the positive correlations between psychopathy and the consumption of red meat and poultry remained significant. Compared to that, in the female subsample, narcissism and psychopathy were negatively linked to the consumption of fruits. Most of the positive correlations between the SD3 scales and the consumption of red meat, fish/sea food and poultry remained significant when only the female subsample was investigated.

## Discussion

The present study was the first to investigate the link between dietary choices and primary emotional systems, and the Dark Triad of personality. Results of Study 1 demonstrated that the vegan/vegetarian diet is associated with higher scores in the primary emotional systems of CARE, SADNESS and spirituality, and lower scores in PLAY. However, please note that the link with CARE got weaker after the sex of participants was considered. There were no significant mean differences between vegans/vegetarians and omnivores on Machiavellianism, narcissism and psychopathy after age and sex were taken into consideration. Moreover, correlation analysis demonstrated that CARE, SADNESS and spirituality were negatively linked to meat consumption. Additionally, high Machiavellianism, narcissism and psychopathy were associated with more frequent meat consumption. These last associations remained significant only in the subsample of female participants after the analyses were split by the sex of participants.

Opposite to the results from Study 1, findings from Study 2 revealed that omnivores reported significantly higher scores on all three Dark Triad scales, compared to participants with a vegan/vegetarian diet. However, after the sex of participants was considered, only the differences with respect to Machiavellianism and narcissism remained significant. Most of the significant positive correlations between the frequency of meat consumption and the scores on the Dark Triad traits, found in Study 1, were replicated in Study 2. Those associations were again found primarily in the subsample of female participants.

As suggested in the introductory part of this article, high CARE scores were associated with having vegan/vegetarian diet, as well as higher consumption of fruits and vegetables and lower consumption of meat (as demonstrated by the correlation analysis). These results support findings on the link between higher empathy and a plant-based diet (Preylo and Arikawa, 2008; Filippi et al., 2010). However, per definition next to feeling empathy, CARE encompasses the actual behavior of taking care of others (including children and animals) (Davis et al., 2003). Moreover, some of the brain regions reported to show higher activation in vegans/vegetarians during the presentation of animal negative valence scenes in an fMRI study (Filippi et al., 2010), were also suggested to serve as a biological foundation for the CARE system (Montag and Panksepp, 2016). However, please note that the differences between vegans/vegetarians and omnivores with respect to CARE got weaker after the sex of participants was considered. Additionally, the negative correlations between CARE and meat consumption remained significant only in the female subsample. Thus, the more active CARE system in females might exert a larger effect on food choices (especially regarding meat eating) in females compared to males.

Vegans/vegetarians demonstrated higher SADNESS scores than omnivores. This result supports findings with respect to the link between high neuroticism and depression, and a vegetarian diet (Forestell and Nezlek, 2018). The present study adds to those findings as it was demonstrated that not negative affect as a whole, but only the SADNESS system was linked to dietary choices. On the other hand, neuroticism is correlated with all three negative emotional systems (SADNESS, FEAR and ANGER; Davis and Panksepp, 2011), making it a less specific personality trait. Please refer to the correlation analysis where ANGER was even positively associated with meat eating. This might also help to explain inconsistent findings with respect to negative emotionality (e.g., Beezhold et al., 2015, reported lower anxiety in male vegetarians as compared to omnivores). Thus, replication of findings in needed. Also, please note that the reported effect for SADNESS was small (${\text{η}}_{p}^{2}$ = 0.01), thus demonstrating that many additional factors also play a role in choosing a particular diet.

With respect to spirituality, the results of the present study supported our hypothesis on a positive relation with the vegan/vegetarian diet (here the reported effect was also small: ${\text{η}}_{p}^{2}$ = 0.01). This result was supported also in the correlation analysis where spirituality demonstrated positive associations with fruit and vegetable consumption, and negative associations with meat consumption. Spirituality was defined as seeking harmony and feeling as one with the universe, and was linked to agreeableness and openness to experiences (Davis et al., 2003). However, interestingly no significant association between SEEKING and the vegetarian diet could be found when comparing the group of vegans/vegetarians with the group of omnivores, although openness to experiences (a rather robust predictor of vegetarian food choices) was reported to be most strongly related to SEEKING among the primary emotional systems (Davis et al., 2003). Please note that some positive associations between SEEKING and the consumption of fruits and vegetables were found in the correlation analysis. Future studies need to further explore this link.

Lastly, the effect of diet on PLAY did not remain significant in the MANOVA after a Bonferroni correction. Also, no significant associations between PLAY and meat consumption could be demonstrated in the correlation analysis. However, descriptively the omnivorous group exhibited higher PLAY scores than the vegan/vegetarian group. Given the positive association between PLAY and extraversion, and the link between high extraversion and high meat consumption (e.g., Pfeiler and Egloff, 2018c), the association between PLAY and dietary choices should be further investigated in future studies.

The Dark Triad of personality was also examined for the first time in the context of food choices. Significant mean differences between vegans/vegetarians and omnivores with respect to the Dark Triad traits were found in Study 1, with omnivores reporting higher Machiavellianism scores (before Bonferroni correction) than vegans/vegetarians, and in Study 2, where omnivores scored higher on all three Dark Triad scales compared to vegans/vegetarians. However, after considering the sex of the participants, only the association between Machiavellianism (${\text{η}}_{p}^{2}$ = 0.02), narcissism (${\text{η}}_{p}^{2}$ = 0.01 before Bonferroni correction) and diet in Study 2 remained significant. This finding adds to studies reporting a link between beliefs in humans’ supremacy and the omnivorous diet (Leite et al., 2018), as well as associations between the Dark Triad traits and negative attitudes and cruelty toward animals (Kavanagh et al., 2013).

On the other hand, the correlation patterns between the Dark Triad traits and the frequency of consumption of meat were similar in both studies. Interestingly, the positive correlations between the Dark Triad traits and the frequency of meat consumption remained significant only in the subsample of female participants with only a few exceptions (e.g. positive correlation between psychopathy and frequency of poultry consumption in the male subsample) (please note that the correlation coefficients were rather low). This is an important observation as sex differences with respect to both, the Dark Triad traits and dietary choices, were demonstrated in numerous studies, with males consistently reporting higher scores on the Dark Triad traits and female vegetarians, outnumbering male vegetarians (Ruby, 2012; Muris et al., 2017). Thus, it is important that future studies employ the same number of male and female participants to further examine those effects. The low number of male vegan/vegetarian participants in the present study hampers the examination of interaction effects between diet and sex.

Even though the results of the MANOVA did not deliver significant mean differences with respect to psychopathy after sex was accounted for, the correlation analysis demonstrated positive correlations between all Dark Triad variables and the consumption of meat. Thus, the question arises if a continuous variable (frequency of consumption of different foods; or least strict – to – most strict vegetarian) is a better measure of dietary choices than a dichotomous variable (vegan/vegetarian vs. omnivorous diet).

It needs to be mentioned that the SD3 scales measure Machiavellianism, non-pathological narcissism and non-pathological psychopathy, thus, these constructs need to be understood as personality traits rather than pathological behaviors. The aim of this study is not to pathologize or put a stigma on particular food/dietary choices. Instead, this research project aimed to investigate additional personality traits next to the often examined Five-factor model to help explain additional variance in dietary choices and, thus, better understand the factors, which might shape our food choices. Since the Dark Triad of personality and the Big Five traits overlap to a certain extent (O’Boyle et al., 2015), future studies, aiming at a direct comparison between the predictive abilities of both models with respect to dietary choices need to be conducted.

Moreover, with respect to the associations found between the primary emotional systems and the Dark Triad traits, future studies might consider examining those associations within a mediation model to predict dietary preferences. In such a complex model, also the role of sex as a moderator in this relationship might be considered. However, due to missing theoretical and empirical evidence regarding the direction of those associations (to the authors’ knowledge this is the first study to examine the link between the primary emotional systems, Dark Triad traits and dietary preferences), we refrain from testing such a model in the present study.

In the following, some of the limitations of the study will be discussed. Even though sex was considered as a potential moderating variable, the number of male participants was much lower than the number of female participants as already discussed earlier in the text. With respect to sex differences, being linked to personality as well as eating style, it is of high importance that future studies employ the same number of male and female participants per group to further examine these relationships (see Ruby and Heine, 2011). Moreover, the subsample of vegans/vegetarians in the present study was rather small. Hence, in future studies the same number of participants per groups should be recruited and samples should be paralleled in order to eliminate possible sociocultural and demographic influences. Additionally, other possible alignments of groups such as the investigation of health-oriented versus ethically oriented vegans and vegetarians might be considered. Furthermore, the importance of investigating vegans and vegetarians separately has been highlighted in previous studies. For example, empirical research revealed higher openness and lower neuroticism scores in vegans compared to vegetarians (Kessler et al., 2016). Last but not least, in the current study the group of pescatarians was not examined for reasons named earlier in the text, however, future studies might consider comparing pescatarians with vegans, vegetarians and omnivores to further disentangle individual differences in personality between these groups.

Lastly, as already mentioned earlier, the assignment of participants to two groups (vegans/vegetarians vs. omnivores) and, thus, the use of a dichotomous variable to test the hypotheses of the study, might be seen as another limitation of the present research project. First, in the present study a rather strict definition of diet was used, since some vegetarians might occasionally consume fish or meat. Thus, future studies might consider assessing dietary choices as a continuous variable, ranging from very strict to least strict vegetarian in order to depict a wider spectrum of dietary styles.

Despite the limitations listed above, the present research project recruited two large samples in order to examine (for the first time) the link between dietary choices and primary emotional systems and the Dark Triad traits. Moreover, Study 2 aimed at the replication of results from Study 1.

## Conclusion

The present study for the first time examined the relationship between dietary choices (vegan/vegetarian vs. omnivorous diet) and the primary emotional systems, and the Dark Triad of personality. High CARE, SADNESS, spirituality and low PLAY were linked to following a vegan/vegetarian diet (Study 1) (please note that some associations got weaker after the sex of participants was considered in the analyses). Additionally, Machiavellianism (Study 1 and 2), narcissism (Study 2) and psychopathy (Study 2) were positively associated with following an omnivorous diet. However, those results got weaker or disappeared after the sex of participants was considered as a second independent variable. The findings of this study add to the literature because until now the focus lied mainly on the Five-factor model of personality. Lastly, the results of this study might help reconsider existing, rather negative stereotypes linked to the choice of a vegan or a vegetarian diet.

## Data Availability Statement

The raw data from Study 1, supporting the conclusion of this manuscript will be made available by the authors, only upon a reasonable request. The raw data from Study 2 is available as a Supplementary Material (“Raw Data Study 2”).

## Author Contributions

RS, BL, and CM designed the study and recruited the participants for the current study. SM contributed protocols and advise. RS conducted the statistical analyses and drafted the first version of the manuscript. All authors worked over the manuscript again and approved its final draft.

## Conflict of Interest

The authors declare that the research was conducted in the absence of any commercial or financial relationships that could be construed as a potential conflict of interest.

## Footnotes

1 The capitalizations are used to specify the primary emotional systems as compared to other commonly used psychological terms (Davis and Panksepp, 2011).

2 Such inconsistencies were tested for vegans/vegetarians and pescatarians. For participants, reporting to not restrict their diet with respect to animal products on question 1 (omnivores), such inconsistency checks were not conducted, because 1) omnivores do not have to consume meat with every meal/every week, 2) people usually recall their behavior during the last couple of weeks (thus, they might report to not consume meat in a regular week, because they have not eaten meat for a couple of weeks), 3) we assume that people, who chose this answer, consciously decide to not restrict their diet with respect to animal products.
