# Supplementary material for: What Does Our Personality Say About Our Dietary Choices? Insights on the Associations Between Dietary Habits, Primary Emotional Systems and the Dark Triad of Personality
Source: Front Psychol. 2019 Nov 22;10:2591. doi: 10.3389/fpsyg.2019.02591 (PMC6883900; doi:10.3389/fpsyg.2019.02591)
Supplement: Supplementary file 2 [file Data_Sheet_2.docx]

**SPSS Syntax for the analyses of the data from study 2**

********SD3 SUM******

RECODE SD3_05 SD3_06 SD3_17 SD3_21 SD3_23 (1=5) (2=4) (3=3) (4=2) (5=1).

EXECUTE.

COMPUTE Machiav_Sum = SUM (SD3_01, SD3_04, SD3_07, SD3_10, SD3_13, SD3_16, SD3_19, SD3_22, SD3_25).

EXECUTE.

COMPUTE Narcis_Sum = SUM (SD3_02, SD3_05, SD3_08, SD3_11, SD3_14, SD3_17, SD3_20, SD3_23, SD3_26).

EXECUTE.

COMPUTE Psychopath_Sum = SUM (SD3_03, SD3_06, SD3_09, SD3_12, SD3_15, SD3_18, SD3_21, SD3_24, SD3_27).

EXECUTE.

RELIABILITY

/VARIABLES=SD3_01 SD3_04 SD3_07 SD3_10 SD3_13 SD3_16 SD3_19 SD3_22 SD3_25

/SCALE('ALL VARIABLES') ALL

/MODEL=ALPHA

/SUMMARY=TOTAL.

RELIABILITY

/VARIABLES=SD3_02 SD3_05 SD3_08 SD3_11 SD3_14 SD3_17 SD3_20 SD3_23 SD3_26

/SCALE('ALL VARIABLES') ALL

/MODEL=ALPHA

/SUMMARY=TOTAL.

RELIABILITY

/VARIABLES=SD3_03 SD3_06 SD3_09 SD3_12 SD3_15 SD3_18 SD3_21 SD3_24 SD3_27

/SCALE('ALL VARIABLES') ALL

/MODEL=ALPHA

/SUMMARY=TOTAL.

RECODE SD3_05 SD3_06 SD3_17 SD3_21 SD3_23 (1=5) (2=4) (3=3) (4=2) (5=1).

EXECUTE.

***************************************************************************************************************************************

**************************Descriptive statistics**************************

RECODE EBQ_01 (1=1) (2=1) (4=2) INTO EBQ_V_O.

EXECUTE.

VALUE LABELS EBQ_V_O 1 'vegan/vegetarian' 2 'omnivore'.

EXECUTE.

FREQUENCIES VARIABLES=Sex Education Age EBQ_V_O

/STATISTICS=STDDEV MAXIMUM MINIMUM MEAN

/ORDER=ANALYSIS.

SORT CASES BY Sex.

SPLIT FILE SEPARATE BY Sex.

FREQUENCIES VARIABLES=EBQ_V_O

/ORDER=ANALYSIS.

SPLIT FILE OFF.

****Filter only the group of vegans/vegetarians for the following analysis.****

USE ALL.

COMPUTE filter_$=(EBQ_V_O = 1).

VARIABLE LABELS filter_$ '(EBQ_V_O = 1) (FILTER)'.

VALUE LABELS filter_$ 0 'Not Selected' 1 'Selected'.

FORMATS filter_$ (f1.0).

FILTER BY filter_$.

EXECUTE.

FREQUENCIES VARIABLES=EBQ_02

/ORDER=ANALYSIS.

FILTER OFF.

USE ALL.

EXECUTE.

*********************Descriptive statistics and associations with age and gender***********************

SORT CASES BY EBQ_V_O.

SPLIT FILE SEPARATE BY EBQ_V_O.

FREQUENCIES VARIABLES=Age Machiav_Sum Narcis_Sum Psychopath_Sum

/STATISTICS=STDDEV MINIMUM MAXIMUM MEAN MEDIAN SKEWNESS SESKEW KURTOSIS SEKURT

/ORDER=ANALYSIS.

SPLIT FILE OFF.

NPAR TESTS

/M-W= Age BY EBQ_V_O(1 2)

/MISSING ANALYSIS.

CORRELATIONS

/VARIABLES=Age Machiav_Sum Narcis_Sum Psychopath_Sum

/PRINT=TWOTAIL NOSIG

/MISSING=PAIRWISE.

CROSSTABS

/TABLES=EBQ_V_O BY Sex

/FORMAT=AVALUE TABLES

/STATISTICS=CHISQ

/CELLS=COUNT

/COUNT ROUND CELL.

FREQUENCIES VARIABLES=Machiav_Sum Narcis_Sum Psychopath_Sum

/STATISTICS=STDDEV MINIMUM MAXIMUM MEAN MEDIAN

/ORDER=ANALYSIS.

SORT CASES BY Sex.

SPLIT FILE SEPARATE BY Sex.

FREQUENCIES VARIABLES=Machiav_Sum Narcis_Sum Psychopath_Sum

/STATISTICS=STDDEV MINIMUM MAXIMUM MEAN MEDIAN

/ORDER=ANALYSIS.

SPLIT FILE OFF.

T-TEST GROUPS=Sex(1 2)

/MISSING=ANALYSIS

/VARIABLES=Machiav_Sum Narcis_Sum Psychopath_Sum

/CRITERIA=CI(.95).

************** MANOVA and correlation analyses**************

GLM Machiav_Sum Narcis_Sum Psychopath_Sum BY EBQ_V_O

/METHOD=SSTYPE(3)

/INTERCEPT=INCLUDE

/PLOT=PROFILE(EBQ_V_O) TYPE=BAR ERRORBAR=NO MEANREFERENCE=NO

/PRINT=DESCRIPTIVE ETASQ

/CRITERIA=ALPHA(.05)

/DESIGN= EBQ_V_O.

GLM Machiav_Sum Narcis_Sum Psychopath_Sum BY EBQ_V_O Sex

/METHOD=SSTYPE(3)

/INTERCEPT=INCLUDE

/PLOT=PROFILE(EBQ_V_O*Sex) TYPE=BAR ERRORBAR=NO MEANREFERENCE=NO

/PRINT=DESCRIPTIVE ETASQ

/CRITERIA=ALPHA(.05)

/DESIGN= EBQ_V_O Sex EBQ_V_O*Sex.

FREQUENCIES VARIABLES=EBQ_03 EBQ_04 EBQ_05 EBQ_06 EBQ_07 EBQ_08 EBQ_09 EBQ_10 EBQ_11 EBQ_12

/STATISTICS=STDDEV MINIMUM MAXIMUM MEAN MEDIAN SKEWNESS SESKEW KURTOSIS SEKURT

/HISTOGRAM NORMAL

/ORDER=ANALYSIS.

BOOTSTRAP

/SAMPLING METHOD=SIMPLE

/VARIABLES INPUT=Machiav_Sum Narcis_Sum Psychopath_Sum EBQ_03 EBQ_04 EBQ_05 EBQ_06 EBQ_07 EBQ_08

EBQ_09 EBQ_10 EBQ_11 EBQ_12

/CRITERIA CILEVEL=95 CITYPE=BCA NSAMPLES=1000

/MISSING USERMISSING=EXCLUDE.

CORRELATIONS

/VARIABLES=Machiav_Sum Narcis_Sum Psychopath_Sum EBQ_03 EBQ_04 EBQ_05 EBQ_06 EBQ_07 EBQ_08 EBQ_09

EBQ_10 EBQ_11 EBQ_12

/PRINT=TWOTAIL NOSIG

/MISSING=PAIRWISE.

BOOTSTRAP

/SAMPLING METHOD=SIMPLE

/VARIABLES INPUT=Machiav_Sum Narcis_Sum Psychopath_Sum EBQ_03 EBQ_04 EBQ_05 EBQ_06 EBQ_07 EBQ_08

EBQ_09 EBQ_10 EBQ_11 EBQ_12

/CRITERIA CILEVEL=95 CITYPE=BCA NSAMPLES=1000

/MISSING USERMISSING=EXCLUDE.

NONPAR CORR

/VARIABLES=Machiav_Sum Narcis_Sum Psychopath_Sum EBQ_03 EBQ_04 EBQ_05 EBQ_06 EBQ_07 EBQ_08 EBQ_09

EBQ_10 EBQ_11 EBQ_12

/PRINT=SPEARMAN TWOTAIL NOSIG

/MISSING=PAIRWISE.

SORT CASES BY Sex.

SPLIT FILE SEPARATE BY Sex.

BOOTSTRAP

/SAMPLING METHOD=SIMPLE

/VARIABLES INPUT=Machiav_Sum Narcis_Sum Psychopath_Sum EBQ_03 EBQ_04 EBQ_05 EBQ_06 EBQ_07 EBQ_08

EBQ_09 EBQ_10 EBQ_11 EBQ_12

/CRITERIA CILEVEL=95 CITYPE=BCA NSAMPLES=1000

/MISSING USERMISSING=EXCLUDE.

CORRELATIONS

/VARIABLES=Machiav_Sum Narcis_Sum Psychopath_Sum EBQ_03 EBQ_04 EBQ_05 EBQ_06 EBQ_07 EBQ_08 EBQ_09

EBQ_10 EBQ_11 EBQ_12

/PRINT=TWOTAIL NOSIG

/MISSING=PAIRWISE.

SPLIT FILE OFF.
