# Supplementary material for: What Does Our Personality Say About Our Dietary Choices? Insights on the Associations Between Dietary Habits, Primary Emotional Systems and the Dark Triad of Personality
Source: Front Psychol. 2019 Nov 22;10:2591. doi: 10.3389/fpsyg.2019.02591 (PMC6883900; doi:10.3389/fpsyg.2019.02591)
Supplement: Supplementary file 3 [file Table_1.docx]

**Supplementary material**

Table S1. Overall and by sex descriptive statistics for the ANPS scales (study 1).

|  | SEEKING | FEAR | CARE | ANGER | PLAY | SADNESS | spirituality |
| --- | --- | --- | --- | --- | --- | --- | --- |
| *N* | 1140 | 1140 | 1140 | 1140 | 1140 | 1140 | 1140 |
| Mean | 39.70 | 36.75 | 41.34 | 35.86 | 42.10 | 34.78 | 25.49 |
| Median | 40 | 37 | 42 | 36 | 42 | 35 | 26 |
| *SD* | 4.45 | 6.66 | 5.89 | 6.19 | 5.57 | 5.34 | 6.46 |
| Min. | 20 | 17 | 16 | 17 | 19 | 20 | 12 |
| Max. | 53 | 54 | 56 | 56 | 56 | 54 | 47 |
| **males** | | | | | | | |
| *n* | 357 | 357 | 357 | 357 | 357 | 357 | 357 |
| Mean | 39.61 | 34.36 | 37.80 | 35.15 | 42.37 | 32.50 | 24.22 |
| Median | 40 | 34 | 38 | 35 | 43 | 32 | 24 |
| *SD* | 4.73 | 6.47 | 5.71 | 6.15 | 5.75 | 5.07 | 6.67 |
| Min. | 20 | 18 | 16 | 17 | 21 | 22 | 12 |
| Max. | 52 | 53 | 53 | 56 | 56 | 49 | 44 |
| **females** | | | | | | | |
| *n* | 783 | 783 | 783 | 783 | 783 | 783 | 783 |
| Mean | 39.75 | 37.83 | 42.96 | 36.19 | 41.98 | 35.81 | 26.07 |
| Median | 40 | 38 | 43 | 36 | 42 | 36 | 26 |
| *SD* | 4.33 | 6.47 | 5.22 | 6.18 | 5.48 | 5.13 | 6.29 |
| Min. | 25 | 17 | 24 | 19 | 19 | 20 | 12 |
| Max. | 53 | 54 | 56 | 54 | 56 | 54 | 47 |
| t test | *t*(1138) =  - .494 | *t*(1138) =  - 8.399 | *t*(1138) =  - 15.044 | *t*(1138) =  - 2.634 | *t*(1138) = 1.087 | *t*(1138) =  - 10.139 | *t*(654.680) =  - 4.440 |
|  | n.s. | *p* < .01 | *p* < .01 | *p* < .01 | n.s. | *p* < .01 | *p* < .01 |

Note. n.s. = not significant, when *p* > .05. SD = standard deviation. Two-tailed tests.

Table S2. Overall and by sex descriptive statistics for the Dark Triad traits (study 1).

|  | Machiavellianism | narcissism | psychopathy |
| --- | --- | --- | --- |
| *N* | 508 | 508 | 508 |
| Mean | 26.42 | 23.42 | 18.61 |
| Median | 26.50 | 23 | 18 |
| *SD* | 5.72 | 4.31 | 5.12 |
| Min. | 12 | 11 | 9 |
| Max. | 44 | 38 | 38 |
| **males** | | | |
| *n* | 158 | 158 | 158 |
| Mean | 28.80 | 24.59 | 21.04 |
| Median | 29 | 25 | 21 |
| *SD* | 5.57 | 4.11 | 5.37 |
| Min. | 12 | 12 | 10 |
| Max. | 44 | 34 | 38 |
| **females** | | | |
| *n* | 350 | 350 | 350 |
| Mean | 25.34 | 22.89 | 17.52 |
| Median | 25 | 23 | 17 |
| *SD* | 5.46 | 4.30 | 4.60 |
| Min. | 12 | 11 | 9 |
| Max. | 38 | 38 | 31 |
| t test | *t*(506) = 6.555 | *t*(506) = 4.166 | *t*(506) = 7.567 |
|  | *p* < .01 | *p* < .01 | *p* < .01 |

Note: *SD* = standard deviation. Two-tailed tests.

Table S3. Bootstrap BCa 95 % CI Pearson correlations on the relationship between ANPS and the consumption of different foods for the complete sample (study 1).

|  | honey | fruits | pork | eggs | pasta/noodles | red meat | fish/ sea food | vegetables | potatoes | poultry |
| --- | --- | --- | --- | --- | --- | --- | --- | --- | --- | --- |
| SEEKING | 0.04 | 0.14^**^ | -0.06^*^ | 0.03 | 0.01 | 0.00 | 0.04 | 0.17^**^ | 0.05 | -0.05 |
| BCa 95 % CI lower | -0.02 | 0.08 | -0.12 | -0.03 | -0.05 | -0.05 | -0.02 | 0.11 | -0.01 | -0.11 |
| upper | 0.09 | 0.20 | 0.00 | 0.09 | 0.07 | 0.06 | 0.10 | 0.23 | 0.11 | 0.00 |
| FEAR | -0.09^**^ | -0.04 | -0.07^*^ | -0.07^*^ | 0.01 | -0.10^**^ | -0.08^**^ | 0.02 | -0.03 | -0.01 |
| BCa 95 % CI lower | -0.14 | -0.10 | -0.12 | -0.12 | -0.05 | -0.16 | -0.14 | -0.04 | -0.08 | -0.08 |
| upper | -0.03 | 0.01 | -0.01 | -0.01 | 0.07 | -0.04 | -0.02 | 0.08 | 0.03 | 0.05 |
| CARE | 0.06 | 0.12^**^ | -0.20^**^ | -0.07^*^ | -0.02 | -0.20^**^ | -0.11^**^ | 0.16^**^ | -0.03 | -0.17^**^ |
| BCa 95 % CI lower | 0.01 | 0.06 | -0.26 | -0.13 | -0.07 | -0.26 | -0.17 | 0.10 | -0.10 | -0.23 |
| upper | 0.11 | 0.17 | -0.13 | -0.01 | 0.04 | -0.13 | -0.05 | 0.22 | 0.03 | -0.10 |
| ANGER | -0.02 | -0.07^*^ | 0.04 | 0.08^*^ | 0.07^*^ | 0.09^**^ | 0.02 | -0.04 | -0.00 | 0.09^**^ |
| BCa 95 % CI lower | -0.07 | -0.13 | -0.02 | 0.01 | 0.01 | 0.03 | -0.04 | -0.10 | -0.06 | 0.02 |
| upper | 0.04 | -0.01 | 0.11 | 0.13 | 0.13 | 0.14 | 0.08 | 0.02 | 0.06 | 0.15 |
| PLAY | 0.05 | 0.04 | 0.06 | 0.07^*^ | 0.06^*^ | -0.03 | -0.00 | 0.01 | -0.02 | 0.01 |
| BCa 95 % CI lower | -0.01 | -0.02 | -0.01 | 0.01 | 0.00 | -0.09 | -0.06 | -0.06 | -0.08 | -0.05 |
| upper | 0.11 | 0.11 | 0.12 | 0.13 | 0.13 | 0.04 | 0.06 | 0.08 | 0.04 | 0.08 |
| SADNESS | -0.07^*^ | -0.01 | -0.11^**^ | -0.07^*^ | 0.02 | -0.12^**^ | -0.10^**^ | 0.04 | -0.03 | -0.05 |
| BCa 95 % CI lower | -0.12 | -0.07 | -0.16 | -0.13 | -0.05 | -0.17 | -0.16 | -0.01 | -0.09 | -0.12 |
| upper | -0.01 | 0.06 | -0.06 | -0.01 | 0.08 | -0.05 | -0.05 | 0.10 | 0.03 | 0.01 |
| spirituality | 0.10^**^ | 0.10^**^ | -0.15^**^ | 0.01 | -0.07^*^ | -0.07^*^ | -0.01 | 0.13^**^ | 0.04 | -0.12^**^ |
| BCa 95 % CI lower | 0.03 | 0.04 | -0.20 | -0.05 | -0.13 | -0.12 | -0.07 | 0.06 | -0.02 | -0.18 |
| upper | 0.16 | 0.16 | -0.09 | 0.07 | -0.01 | -0.02 | 0.05 | 0.18 | 0.10 | -0.06 |

Note: *N* = 1140, ^*^*p* < .05, ^**^*p* < .01; two-tailed tests. Bootstrap results are based on 1000 bootstrap samples.

Table S4. Bootstrap BCa 95 % CI Pearson correlations on the relationship between the ANPS scales and the consumption of different foods split by sex (study 1).

| **males** | | | | | | | | | | |
| --- | --- | --- | --- | --- | --- | --- | --- | --- | --- | --- |
|  | honey | fruits | pork | eggs | pasta/noodles | red meat | fish/ sea food | vegetables | potatoes | poultry |
| SEEKING | 0.05 | 0.11^*^ | -0.09 | 0.15^**^ | 0.02 | -0.04 | -0.02 | 0.19^**^ | 0.06 | -0.05 |
| BCa 95 % CI lower | -0.07 | 0.00 | -0.19 | 0.04 | -0.08 | -0.14 | -0.12 | 0.09 | -0.04 | -0.14 |
| upper | 0.17 | 0.21 | 0.00 | 0.25 | 0.12 | 0.05 | 0.08 | 0.29 | 0.16 | 0.05 |
| FEAR | -0.12^*^ | -0.13^*^ | 0.08 | -0.08 | 0.06 | -0.00 | -0.10 | -0.02 | 0.01 | 0.05 |
| BCa 95 % CI lower | -0.21 | -0.22 | -0.01 | -0.18 | -0.05 | -0.12 | -0.19 | -0.12 | -0.10 | -0.08 |
| Upper | -0.02 | -0.04 | 0.18 | 0.03 | 0.17 | 0.12 | 0.00 | 0.10 | 0.12 | 0.17 |
| CARE | 0.07 | 0.01 | -0.10 | 0.09 | -0.03 | -0.08 | -0.04 | 0.08 | 0.01 | -0.09 |
| BCa 95 % CI lower | -0.02 | -0.09 | -0.22 | -0.02 | -0.14 | -0.19 | -0.13 | -0.04 | -0.10 | -0.19 |
| upper | 0.16 | 0.10 | 0.01 | 0.19 | 0.06 | 0.02 | 0.06 | 0.18 | 0.12 | 0.00 |
| ANGER | -0.12^*^ | -0.07 | 0.07 | 0.04 | 0.08 | 0.15^**^ | -0.01 | -0.03 | 0.02 | 0.18^**^ |
| BCa 95 % CI lower | -0.24 | -0.17 | -0.04 | -0.07 | -0.02 | 0.04 | -0.12 | -0.15 | -0.09 | 0.07 |
| upper | -0.01 | 0.03 | 0.17 | 0.16 | 0.18 | 0.26 | 0.09 | 0.09 | 0.12 | 0.29 |
| PLAY | 0.02 | 0.05 | 0.03 | 0.15^**^ | 0.01 | -0.03 | -0.06 | 0.02 | 0.03 | -0.01 |
| BCa 95 % CI lower | -0.08 | -0.05 | -0.08 | 0.04 | -0.09 | -0.15 | -0.19 | -0.09 | -0.09 | -0.12 |
| upper | 0.11 | 0.15 | 0.15 | 0.24 | 0.11 | 0.09 | 0.08 | 0.12 | 0.13 | 0.11 |
| SADNESS | -0.14^**^ | -0.09 | 0.01 | -0.05 | 0.06 | 0.01 | -0.14^**^ | 0.00 | 0.02 | -0.01 |
| BCa 95 % CI lower | -0.24 | -0.19 | -0.09 | -0.14 | -0.05 | -0.11 | -0.24 | -0.10 | -0.08 | -0.13 |
| upper | -0.05 | 0.00 | 0.10 | 0.06 | 0.17 | 0.12 | -0.04 | 0.11 | 0.13 | 0.11 |
| spirituality | 0.16^**^ | 0.11^*^ | -0.19^**^ | 0.07 | -0.05 | -0.13^*^ | 0.05 | 0.15^**^ | 0.04 | -0.12^*^ |
| BCa 95 % CI lower | 0.05 | 0.01 | -0.29 | -0.04 | -0.16 | -0.22 | -0.06 | 0.05 | -0.06 | -0.24 |
| upper | 0.26 | 0.21 | -0.09 | 0.17 | 0.05 | -0.04 | 0.14 | 0.23 | 0.14 | -0.01 |

| **females** | | | | | | | | | | |
| --- | --- | --- | --- | --- | --- | --- | --- | --- | --- | --- |
|  | honey | fruits | pork | eggs | pasta/noodles | red meat | fish/ sea food | vegetables | potatoes | poultry |
| SEEKING | 0.03 | 0.16^**^ | -0.04 | -0.03 | 0.01 | 0.04 | 0.07^*^ | 0.16^**^ | 0.05 | -0.05 |
| BCa 95 % CI lower | -0.04 | 0.09 | -0.11 | -0.09 | -0.06 | -0.04 | 0.00 | 0.09 | -0.04 | -0.12 |
| upper | 0.10 | 0.22 | 0.03 | 0.04 | 0.09 | 0.11 | 0.14 | 0.22 | 0.14 | 0.02 |
| FEAR | -0.08^*^ | -0.08^*^ | -0.02 | -0.01 | 0.02 | -0.04 | -0.03 | -0.06 | -0.00 | 0.04 |
| BCa 95 % CI lower | -0.14 | -0.14 | -0.10 | -0.09 | -0.06 | -0.10 | -0.09 | -0.13 | -0.07 | -0.04 |
| upper | -0.01 | 0.00 | 0.07 | 0.06 | 0.09 | 0.02 | 0.03 | 0.01 | 0.06 | 0.11 |
| CARE | 0.05 | 0.06 | -0.03 | -0.06 | 0.04 | -0.08^*^ | -0.08^*^ | 0.06 | 0.01 | -0.09^**^ |
| BCa 95 % CI lower | -0.01 | -0.02 | -0.10 | -0.13 | -0.02 | -0.15 | -0.15 | -0.02 | -0.06 | -0.16 |
| upper | 0.11 | 0.12 | 0.03 | 0.00 | 0.11 | -0.01 | 0.01 | 0.13 | 0.08 | -0.02 |
| ANGER | 0.03 | -0.09^*^ | 0.08^*^ | 0.11^**^ | 0.07^*^ | 0.10^**^ | 0.05 | -0.07^*^ | 0.00 | 0.07 |
| BCa 95 % CI lower | -0.03 | -0.16 | 0.00 | 0.04 | 0.00 | 0.02 | -0.02 | -0.14 | -0.08 | -0.01 |
| upper | 0.09 | -0.02 | 0.16 | 0.18 | 0.14 | 0.17 | 0.12 | 0.00 | 0.08 | 0.13 |
| PLAY | 0.07 | 0.05 | 0.06 | 0.02 | 0.08^*^ | -0.04 | 0.02 | 0.02 | -0.04 | 0.02 |
| BCa 95 % CI lower | 0.00 | -0.03 | -0.01 | -0.05 | 0.01 | -0.12 | -0.05 | -0.05 | -0.13 | -0.05 |
| upper | 0.13 | 0.12 | 0.13 | 0.09 | 0.16 | 0.03 | 0.09 | 0.08 | 0.04 | 0.08 |
| SADNESS | -0.04 | -0.06 | -0.02 | -0.02 | 0.04 | -0.05 | -0.03 | -0.05 | -0.01 | 0.01 |
| BCa 95 % CI lower | -0.10 | -0.13 | -0.10 | -0.09 | -0.03 | -0.12 | -0.10 | -0.12 | -0.08 | -0.06 |
| upper | 0.02 | 0.02 | 0.06 | 0.05 | 0.10 | 0.03 | 0.04 | 0.03 | 0.06 | 0.09 |
| spirituality | 0.07 | 0.05 | -0.05 | 0.01 | -0.07 | 0.03 | -0.01 | 0.07 | 0.06 | -0.08^*^ |
| BCa 95 % CI lower | 0.00 | -0.03 | -0.13 | -0.05 | -0.13 | -0.05 | -0.08 | 0.00 | -0.01 | -0.15 |
| upper | 0.14 | 0.13 | 0.04 | 0.08 | 0.00 | 0.10 | 0.07 | 0.13 | 0.12 | 0.00 |

*N*(males) = 357, *n*(females) = 783, ^*^*p* < .05, ^**^*p* < .01, two-tailed tests. Bootstrap results are based on 1000 bootstrap samples.

Table S5. Bootstrap BCa 95 % CI Pearson correlations on the relationship between the Dark Triad traits and the consumption of different foods for the complete sample (study 1).

|  | honey | fruits | pork | eggs | pasta/noodles | red meat | fish/ sea food | vegetables | potatoes | poultry |
| --- | --- | --- | --- | --- | --- | --- | --- | --- | --- | --- |
| Machiavellianism | -0.03 | -0.10^*^ | 0.15^**^ | 0.06 | 0.08 | 0.17^**^ | 0.09^*^ | -0.19^**^ | 0.06 | 0.17^**^ |
| BCa 95 % CI lower | -0.11 | -0.19 | 0.06 | -0.03 | -0.01 | 0.09 | 0.00 | -0.27 | -0.03 | 0.07 |
| upper | 0.05 | 0.00 | 0.25 | 0.16 | 0.17 | 0.24 | 0.18 | -0.10 | 0.15 | 0.26 |
| narcissism | 0.12^**^ | -0.01 | 0.08 | 0.12^**^ | 0.06 | 0.15^**^ | 0.17^**^ | -0.05 | 0.04 | 0.15^**^ |
| BCa 95 % CI lower | 0.01 | -0.10 | -0.02 | 0.02 | -0.02 | 0.05 | 0.08 | -0.14 | -0.05 | 0.05 |
| upper | 0.21 | 0.08 | 0.17 | 0.21 | 0.14 | 0.25 | 0.25 | 0.06 | 0.14 | 0.24 |
| psychopathy | -0.02 | -0.12^**^ | 0.15^**^ | 0.11^*^ | 0.04 | 0.18^**^ | 0.09 | -0.10^*^ | -0.01 | 0.18^**^ |
| BCa 95 % CI lower | -0.10 | -0.21 | 0.05 | 0.01 | -0.04 | 0.10 | 0.00 | -0.18 | -0.10 | 0.08 |
| upper | 0.08 | -0.02 | 0.25 | 0.20 | 0.13 | 0.26 | 0.18 | 0.00 | 0.08 | 0.27 |

Note: *N* = 508, ^*^*p* < .05, ^**^*p* < .01, two-tailed tests. Bootstrap results are based on 1000 bootstrap samples.

Table S6. Bootstrap BCa 95 % CI Pearson correlations on the relationship between the Dark Triad traits and the consumption of different foods split in males and females (study 1).

| **males** | | | | | | | | | | |
| --- | --- | --- | --- | --- | --- | --- | --- | --- | --- | --- |
|  | honey | fruits | pork | eggs | pasta/noodles | red meat | fish/ sea food | vegetables | potatoes | poultry |
| Machiavellianism | -0.08 | 0.03 | -0.01 | -0.01 | 0.02 | 0.07 | -0.00 | -0.09 | 0.08 | 0.10 |
| BCa 95 % CI lower | -0.23 | -0.13 | -0.17 | -0.18 | -0.17 | -0.12 | -0.17 | -0.24 | -0.09 | -0.07 |
| upper | 0.08 | 0.19 | 0.17 | 0.16 | 0.19 | 0.23 | 0.16 | 0.07 | 0.25 | 0.27 |
| narcissism | 0.09 | 0.07 | -0.11 | 0.12 | -0.01 | -0.06 | 0.07 | -0.04 | 0.01 | 0.08 |
| BCa 95 % CI lower | -0.08 | -0.09 | -0.27 | -0.07 | -0.19 | -0.26 | -0.10 | -0.20 | -0.16 | -0.10 |
| upper | 0.28 | 0.22 | 0.06 | 0.32 | 0.16 | 0.15 | 0.24 | 0.13 | 0.18 | 0.26 |
| psychopathy | -0.14 | 0.08 | -0.05 | 0.01 | -0.08 | 0.13 | -0.04 | -0.01 | 0.03 | 0.19^*^ |
| BCa 95 % CI lower | -0.28 | -0.07 | -0.22 | -0.17 | -0.22 | -0.01 | -0.18 | -0.19 | -0.11 | 0.03 |
| upper | 0.02 | 0.24 | 0.14 | 0.18 | 0.07 | 0.29 | 0.12 | 0.16 | 0.18 | 0.36 |
| **females** | | | | | | | | | | |
|  | honey | fruits | pork | eggs | pasta/noodles | red meat | fish/ sea food | vegetables | potatoes | poultry |
| Machiavellianism | 0.00 | -0.09 | 0.11^*^ | 0.05 | 0.09 | 0.11^*^ | 0.07 | -0.16^**^ | 0.03 | 0.13^*^ |
| BCa 95 % CI lower | -0.08 | -0.20 | 0.01 | -0.04 | -0.01 | 0.01 | -0.02 | -0.27 | -0.08 | 0.03 |
| upper | 0.08 | 0.00 | 0.22 | 0.14 | 0.19 | 0.21 | 0.15 | -0.05 | 0.13 | 0.23 |
| narcissism | 0.14^*^ | 0.00 | 0.09 | 0.09 | 0.08 | 0.19^**^ | 0.18^**^ | 0.01 | 0.04 | 0.13^*^ |
| BCa 95 % CI lower | 0.02 | -0.10 | -0.01 | -0.01 | -0.02 | 0.09 | 0.06 | -0.10 | -0.07 | 0.01 |
| upper | 0.26 | 0.10 | 0.18 | 0.19 | 0.18 | 0.30 | 0.29 | 0.13 | 0.15 | 0.24 |
| psychopathy | 0.06 | -0.14^**^ | 0.11^*^ | 0.11^*^ | 0.08 | 0.09 | 0.08 | -0.03 | -0.06 | 0.08 |
| BCa 95 % CI lower | -0.03 | -0.24 | 0.00 | 0.01 | -0.02 | -0.02 | -0.01 | -0.14 | -0.16 | -0.01 |
| upper | 0.15 | -0.03 | 0.22 | 0.22 | 0.18 | 0.20 | 0.16 | 0.08 | 0.05 | 0.18 |

Note: *N*(males) = 158, *n*(females) = 350, ^*^*p* < .05, ^**^*p* < .01, two-tailed tests. Bootstrap results are based on 1000 bootstrap samples.

Table S7. Spearman correlations for the variables honey, red meat, fish/sea food and the ANPS scales (study 1).

|  | SEEKING | FEAR | CARE | ANGER | PLAY | SADNESS | spirituality |
| --- | --- | --- | --- | --- | --- | --- | --- |
| honey | 0.03 | -0.09^**^ | 0.07^*^ | -0.01 | 0.07^*^ | -0.05 | 0.11^**^ |
| red meat | 0.01 | -0.11^**^ | -0.22^**^ | 0.08^**^ | -0.01 | -0.15^**^ | -0.08^*^ |
| fish/sea food | 0.04 | -0.08^*^ | -0.10^**^ | 0.02 | 0.02 | -0.10^**^ | -0.01 |

*N* = 1140, ^*^*p* < .05, ^**^*p* < .01, two-tailed tests.

Table S8. Descriptive statistics for the Dark Triad traits for the overall sample and separately for males and females. The raw data is available as a Supplementary file („Raw data Study 2”).

|  | Machiavellianism | narcissism | psychopathy |
| --- | --- | --- | --- |
| *N* | 444 | 444 | 444 |
| Mean | 24.05 | 22.89 | 17.11 |
| Median | 24 | 23 | 16 |
| *SD* | 5.15 | 4.27 | 4.96 |
| Min. | 10 | 12 | 9 |
| Max. | 41 | 37 | 32 |
| **males** | | | |
| *n* | 132 | 132 | 132 |
| Mean | 26.40 | 24.33 | 19.78 |
| Median | 26.50 | 24 | 20 |
| *SD* | 5.22 | 4.33 | 5.22 |
| Min. | 14 | 15 | 11 |
| Max. | 37 | 37 | 32 |
| **females** | | | |
| *n* | 312 | 312 | 312 |
| Mean | 23.05 | 22.29 | 15.98 |
| Median | 23 | 22 | 15 |
| *SD* | 4.79 | 4.11 | 4.39 |
| Min. | 10 | 12 | 9 |
| Max. | 41 | 33 | 31 |
| t test | *t*(442) = 6.557 | *t*(442) = 4.709 | *t*(213.051) = 7.334 |
|  | *p* < .01 | *p* < .01 | *p* < .01 |

Note: *SD* = standard deviation. Two-tailed tests.

Table S9. Bootstrap BCa 95 % Pearson correlations on the associations between the Dark Triad traits and the consumption of different foods for the complete sample. The raw data is available as a Supplementary file („Raw data Study 2”).

|  | honey | fruits | pork | eggs | pasta/ noodles | red meat | fish/sea food | vegetables | potatoes | poultry |
| --- | --- | --- | --- | --- | --- | --- | --- | --- | --- | --- |
| Machiavellianism | 0.04 | -0.14^**^ | 0.15^**^ | 0.12^*^ | -0.00 | 0.20^**^ | 0.13^**^ | -0.15^**^ | 0.06 | 0.19^**^ |
| BCa 95 % CI Lower | -0.07 | -0.24 | 0.07 | 0.02 | -0.11 | 0.12 | 0.04 | -0.24 | -0.02 | 0.10 |
| Upper | 0.13 | -0.02 | 0.24 | 0.22 | 0.09 | 0.29 | 0.20 | -0.06 | 0.15 | 0.28 |
| narcissism | 0.00 | -0.14^**^ | 0.12^**^ | 0.10^*^ | -0.03 | 0.19^**^ | 0.22^**^ | -0.04 | -0.06 | 0.18^**^ |
| BCa 95 % CI Lower | -0.10 | -0.23 | 0.04 | -0.00 | -0.10 | 0.10 | 0.13 | -0.13 | -0.15 | 0.07 |
| Upper | 0.10 | -0.03 | 0.21 | 0.22 | 0.06 | 0.28 | 0.30 | 0.04 | 0.03 | 0.28 |
| psychopathy | -0.04 | -0.19^**^ | 0.18^**^ | 0.11^*^ | 0.06 | 0.21^**^ | 0.15^**^ | -0.16^**^ | 0.06 | 0.22^**^ |
| BCa 95 % CI Lower | -0.13 | -0.29 | 0.08 | 0.01 | -0.04 | 0.12 | 0.06 | -0.26 | -0.04 | 0.12 |
| Upper | 0.04 | -0.09 | 0.28 | 0.21 | 0.17 | 0.32 | 0.23 | -0.07 | 0.15 | 0.31 |

Note: *N* = 444, ^*^*p* < .05, ^**^*p* < .01, two-tailed tests. Bootstrap results are based on 1000 bootstrap samples.

Table S10. Bootstrap BCa 95 % Pearson correlations on the associations between the Dark Triad traits and the consumption of different foods, split in males and females. The raw data is available as a Supplementary file („Raw data Study 2”).

| **males** | | | | | | | | | | |
| --- | --- | --- | --- | --- | --- | --- | --- | --- | --- | --- |
|  | honey | fruits | pork | eggs | pasta/ noodles | red meat | fish/sea food | vegetables | potatoes | poultry |
| Machiavellianism | 0.03 | -0.08 | 0.11 | 0.09 | -0.12 | 0.08 | 0.06 | -0.08 | 0.02 | 0.13 |
| BCa 95 % CI Lower | -0.12 | -0.23 | -0.05 | -0.10 | -0.29 | -0.09 | -0.14 | -0.23 | -0.14 | -0.05 |
| Upper | 0.18 | 0.08 | 0.25 | 0.26 | 0.09 | 0.25 | 0.24 | 0.09 | 0.19 | 0.29 |
| narcissism | 0.00 | 0.01 | -0.00 | 0.08 | -0.04 | 0.09 | 0.14 | 0.11 | 0.04 | 0.15 |
| BCa 95 % CI Lower | -0.17 | -0.16 | -0.14 | -0.11 | -0.20 | -0.07 | -0.08 | -0.05 | -0.12 | -0.05 |
| Upper | 0.20 | 0.20 | 0.15 | 0.29 | 0.15 | 0.24 | 0.34 | 0.26 | 0.18 | 0.32 |
| psychopathy | -0.17 | -0.11 | 0.10 | 0.08 | 0.07 | 0.19^*^ | 0.10 | -0.08 | 0.07 | 0.24^**^ |
| BCa 95 % CI Lower | -0.31 | -0.27 | -0.09 | -0.10 | -0.11 | 0.00 | -0.07 | -0.24 | -0.10 | 0.09 |
| Upper | -0.00 | 0.06 | 0.28 | 0.27 | 0.25 | 0.38 | 0.25 | 0.08 | 0.26 | 0.38 |
| **females** | | | | | | | | | | |
|  | honey | fruits | pork | eggs | pasta/noodles | red meat | fish/sea food | vegetables | potatoes | poultry |
| Machiavellianism | 0.03 | -0.08 | 0.05 | 0.07 | -0.00 | 0.14^*^ | 0.08 | -0.02 | 0.06 | 0.15^**^ |
| BCa 95 % CI Lower | -0.10 | -0.19 | -0.05 | -0.03 | -0.11 | 0.05 | -0.03 | -0.13 | -0.03 | 0.04 |
| Upper | 0.15 | 0.03 | 0.15 | 0.18 | 0.10 | 0.23 | 0.18 | 0.09 | 0.16 | 0.27 |
| narcissism | -0.01 | -0.15^**^ | 0.11 | 0.06 | -0.05 | 0.16^**^ | 0.20^**^ | 0.02 | -0.12^*^ | 0.15^**^ |
| BCa 95 % CI Lower | -0.12 | -0.28 | 0.00 | -0.04 | -0.16 | 0.04 | 0.10 | -0.09 | -0.23 | 0.03 |
| Upper | 0.11 | -0.01 | 0.20 | 0.17 | 0.05 | 0.30 | 0.31 | 0.12 | -0.02 | 0.27 |
| psychopathy | 0.00 | -0.14^*^ | 0.08 | 0.05 | 0.01 | 0.08 | 0.09 | -0.01 | 0.03 | 0.14^*^ |
| BCa 95 % CI Lower | -0.10 | -0.25 | -0.04 | -0.06 | -0.11 | -0.03 | -0.02 | -0.12 | -0.09 | 0.02 |
| Upper | 0.11 | -0.02 | 0.20 | 0.14 | 0.13 | 0.21 | 0.18 | 0.10 | 0.15 | 0.27 |

Note: *n*(male) = 132, *n*(female) = 312, ^*^*p* < .05, ^**^*p* < .01, two-tailed tests. Bootstrap results are based on 1000 bootstrap samples.
